# Restrictive versus standard intravenous fluid therapy and NTproBNP in ICU patients with septic shock – a sub-study of the randomised CLASSIC trial

**DOI:** 10.1186/s12871-026-03836-6

**Published:** 2026-04-15

**Authors:** Jens Christensen, Praleene Sivapalan, Tine Sylvest Meyhoff, Hans Järnbert-Pettersson, Anders Perner, Morten Hylander Møller, Theis Lange, Peter Buhl Hjortrup, Eva Joelsson-Alm, Sandra Jonmarker, Fredric Sjöberg, Johan Mårtensson, Anna Håkansson Gladh, Maria Cronhjort

**Affiliations:** 1 Department of Clinical Science and Education, Södersjukhuset, Karolinska Institutet, Stockholm, Sweden; 2 Copenhagen University Hospital – Rigshospitalet, Copenhagen, Denmark; 3 University of Copenhagen, Section of Biostatistics, Copenhagen, Denmark; 4 Department of Physiology and Pharmacology, Section of Anaesthesia and Intensive Care, Karolinska Institutet, Stockholm, Sweden; 5 Department of Clinical Sciences, Danderyd Hospital, Anaesthesia and Intensive Care at KI DS, Stockholm, Sweden

**Keywords:** Septic shock, Cardiac dysfunction, Fluid therapy, NTproBNP, Randomised controlled trial

## Abstract

**Background:**

Cardiac dysfunction is common in septic shock and may be influenced by intravenous (IV) fluid therapy. We aimed to investigate the effects of restrictive versus standard IV fluid therapy on plasma N-terminal pro B-type natriuretic peptide (NTproBNP), a marker of cardiac dysfunction, in adult intensive care unit (ICU) patients with septic shock.

**Methods:**

This prospective exploratory sub-study of the randomised CLASSIC trial enrolled adult ICU patients with septic shock at one Danish and one Swedish ICU between February 2020 and October 2021. Patients were randomised to restrictive versus standard IV fluid therapy. Plasma NTproBNP was measured at randomisation (T0), the following morning (T1), the morning after (T2), and at ICU discharge or day 90 (T3). The exploratory outcome was the between-group difference in NTproBNP across timepoints assessed using mixed models.

**Results:**

Fifty-four patients were included. NTproBNP levels did not differ significantly between groups at any timepoint. At T1, mean NTproBNP levels were 7667 ng/L in the restrictive group and 8913 ng/L in the standard group, with an estimated between-group difference of -1245 (95% CI -4065–1575). Similarly, no statistically significant differences were observed at T2 and T3. No group-by-time interaction was observed (*p* = 0.925).

**Conclusions:**

No statistically significant difference in plasma NTproBNP between adult ICU patients with septic shock randomised to restrictive versus standard IV fluid therapy was seen. Uniformly elevated levels could suggest that NTproBNP in septic shock primarily reflects systemic inflammation rather than fluid-induced cardiac loading. Results should be interpreted with caution due to the small sample size.

**Trial registration:**

ClinicalTrials.gov NCT04282252. Registered 31 January 2020.

**Supplementary Information:**

The online version contains supplementary material available at 10.1186/s12871-026-03836-6.

## Introduction

Cardiac dysfunction is a common contributor to circulatory failure in patients with septic shock, either by decompensation of preexisting cardiac disease or induction of septic cardiomyopathy (SCM) [1, 2]. SCM is defined as an acute, reversible depression of cardiac contractility and ventricular function induced by sepsis, in the absence of preexisting structural heart disease [3]. Proposed mechanisms include altered loading conditions, myocardial ischemia and increased levels of proinflammatory mediators [4]. SCM occurs in approximately 20% of patients with sepsis and is associated with increased mortality [5].

Current Surviving Sepsis Campaign guidelines recommend an initial intravenous (IV) fluid bolus of 30 mL/kg in sepsis [6]. While increased preload can improve contractility in the normally functioning heart by the Frank-Starling mechanism [7], impaired systolic or diastolic function may limit this response, making excessive fluid administration potentially harmful by increasing myocardial load [4, 8]. Notably, up to 50% of septic patients fail to increase stroke volume in response to a fluid bolus, suggesting that the benefit of liberal fluid therapy may depend on the absence of sepsis-related or pre-existing cardiac dysfunction [9, 10].

N-terminal pro B-type Natriuretic Peptide (NTproBNP), released from myocardial cells in response to increased wall strain, reflects such increases in myocardial load, which may arise from both cardiac dysfunction and fluid overload. Rising filling pressures shift the heart toward a less favourable portion of the Frank Starling curve, resulting in increased wall tension and increases in circulating NTproBNP concentrations [11, 12]. Elevated NTproBNP levels have been associated with diastolic and systolic dysfunction as well as disease severity and mortality in sepsis, although the relationship between NTproBNP and cardiac dysfunction appears less consistent than in non-septic populations [13–18]. Observational studies have also associated greater positive net fluid balance with early rises in NTproBNP in critically ill patients [19]. However, potential causal relationships between IV fluid resuscitation strategies and markers of cardiac load, such as NTproBNP, in septic shock have not previously been assessed in randomised clinical trials.

In the international randomised clinical trial Conservative versus Liberal Approach to Fluid Therapy of Septic Shock in Intensive Care (CLASSIC), adult patients with septic shock were randomised to restrictive versus standard IV fluid therapy [20]. In this exploratory sub-study of the CLASSIC trial, we aimed to assess whether restrictive versus standard fluid therapy was associated with between-group differences in change of NTproBNP during ICU stay.

## Method

### Trial design

This study was conducted as a prospective sub-study of the CLASSIC trial (NCT04282252). The CLASSIC trial was a European, parallel-group, open-label, multicentre, randomised clinical trial in which 1554 ICU patients with septic shock were allocated to restrictive or standard IV fluid therapy [20]. A detailed statistical analysis plan for the present sub-study was published prior to data analysis [21]. This analysis plan differed from the ClinicalTrial.gov registration following a later decision to divide the original project into two separate studies. During data processing, it was discovered that the troponin T assay used targeted Troponin T type 1 (TNNT1) rather than the cardiac-specific isoform Troponin T type (TNNT2). Consequently, troponin T measurements were not specific to myocardial tissue and were excluded from further analysis. We therefore only report exploratory analyses of NTproBNP across the randomised trial groups. This study is reported in accordance with the CONSORT guidelines.

### Participants

Patients eligible for inclusion in this sub-study were those enrolled in the CLASSIC trial at Södersjukhuset, Stockholm, Sweden and Rigshospitalet, Copenhagen, Denmark between February 2nd 2020 and October 22nd 2021. The CLASSIC trial included adult ICU patients with septic shock defined according to the Sepsis-3 criteria [22], who had received at least 1 L of IV fluids in the 24 h prior to screening. Exclusion criteria were septic shock for more than 12 h prior to screening, life-threatening bleeding, acute burn injury affecting > 10% of total body surface area, pregnancy or inability to obtain informed consent [20, 23]. All participants provided consent to take part in both the CLASSIC trial and this sub-study. Inclusion in the present sub-study additionally depended on the availability of on-site staff to perform blood sampling and sample handling.

### Interventions

Participants were randomised to receive either restrictive or standard fluid therapy for a maximum duration of 90 days during ICU admission. In the restrictive fluid group, IV fluids were permitted only in the presence of one the following conditions: (1) severe hypoperfusion according to prespecified criteria, (2) documented fluid losses, (3) dehydration or electrolyte disturbances requiring correction or (4) the need to maintain a daily fluid intake of 1 L [23]. If any of these conditions were met, a bolus of 250–500 mL crystalloid solution could be administered intravenously. Participants in the standard fluid group were to be managed according to the Surviving Sepsis Campaign guidelines, and no upper limit for IV fluid administration was imposed [24].

### Outcomes

The exploratory outcome of this study was the between-group difference in change in plasma NTproBNP concentrations across the two intervention groups at timepoints T0 to T3 as defined below.

### Sample size

The initial sample size calculation was based on hsTnT measurements, where a conservative estimate assuming a standard deviation of 40 ng/L indicated that 104 patients would be required to achieve 80% statistical power to detect a between-group difference in change of 22 ng/L from T0 to T1 with a two-sided alpha of 0.05.

### Randomisation

Randomisation in the CLASSIC trial was carried out using a centralised, computer-generated allocation sequence stratified according to trial site and the presence of metastatic or hematologic malignancy. Patients were randomly assigned in a 1:1 ratio, using permuted blocks of 6 or 8, to either restrictive or standard fluid therapy. Blinding was not applied to participants, treating clinicians, or investigators; however, treatment allocation was concealed from the data and safety monitoring committee as well as trial statisticians [20, 23].

### Blood sampling and analysis

Blood samples were obtained from participants at four predefined timepoints: within the first hour following randomisation in the CLASSIC trial (T0), the next morning (T1), the subsequent morning (T2) and at ICU discharge up to 90 days (T3). At each timepoint, one 5 mL EDTA tube was collected. Samples were drawn either arterially or venously via existing catheters or by direct puncture of a blood vessel. Centrifuged was performed within 3 h of sampling. Following centrifugation, plasma was aliquoted into 5 smaller tubes per timepoint and stored in a research freezer at -70 °C or below. Samples were later transported for analysis at a central laboratory at Södersjukhuset. All procedures for blood sampling and handling was followed a predefined protocol. NTproBNP concentrations were measured using an ELISA assay (Biotechne, DY3604-05).

### Statistical methods

The mean difference in change in NTproBNP concentrations between the restrictive and standard fluid groups across all timepoints were analysed using a mixed-effects linear model. We (a) estimated mixed models including a random intercept and fixed effects for time and treatment group (restrictive vs. standard fluid group) to assess between-group differences. Covariance structures between the timepoints was modelled using a first-order autoregressive model (AR [1]). To determine whether between-group differences varied over time, we (b) evaluated the interaction between time and treatment group. To explore associations between NTproBNP levels and cumulative fluid balance irrespective of randomisation groups, Pearson’s correlation coefficients were calculated between NTproBNP and cumulative fluid balance at T1 and T3, as well as between corresponding measurements of each variable across the two timepoints. Scatter plots were generated with fitted linear regression lines based on ordinary least squares to illustrate these associations. Normality was assessed visually, and Pearson’s r was applied to approximately normally distributed variables. The exploratory outcome is presented as estimated means and between-group differences derived from mixed models, whereas unadjusted (raw) data are displayed separately. Two-tailed p values < 0.05 were considered statistically significant. All statistical analysis were performed using IBM SPSS Statistics 2024 except for the generation of Fig. 3 and multiple imputation analysis which was created through R version 4.3.2 (R Core Team, R Foundation for Statistical Computing).

### Declaration of generative AI and AI-assisted technologies in the manuscript preparation process

During the preparation of this work the authors used the large language model ChatGPT (OpenAI, San Francisco, CA, USA) in order to assist with language refinement. After using this tool/service, the authors reviewed and edited the content as needed and take full responsibility for the content of the published article.

## Results

### Patient characteristics

**Fig. 1:**
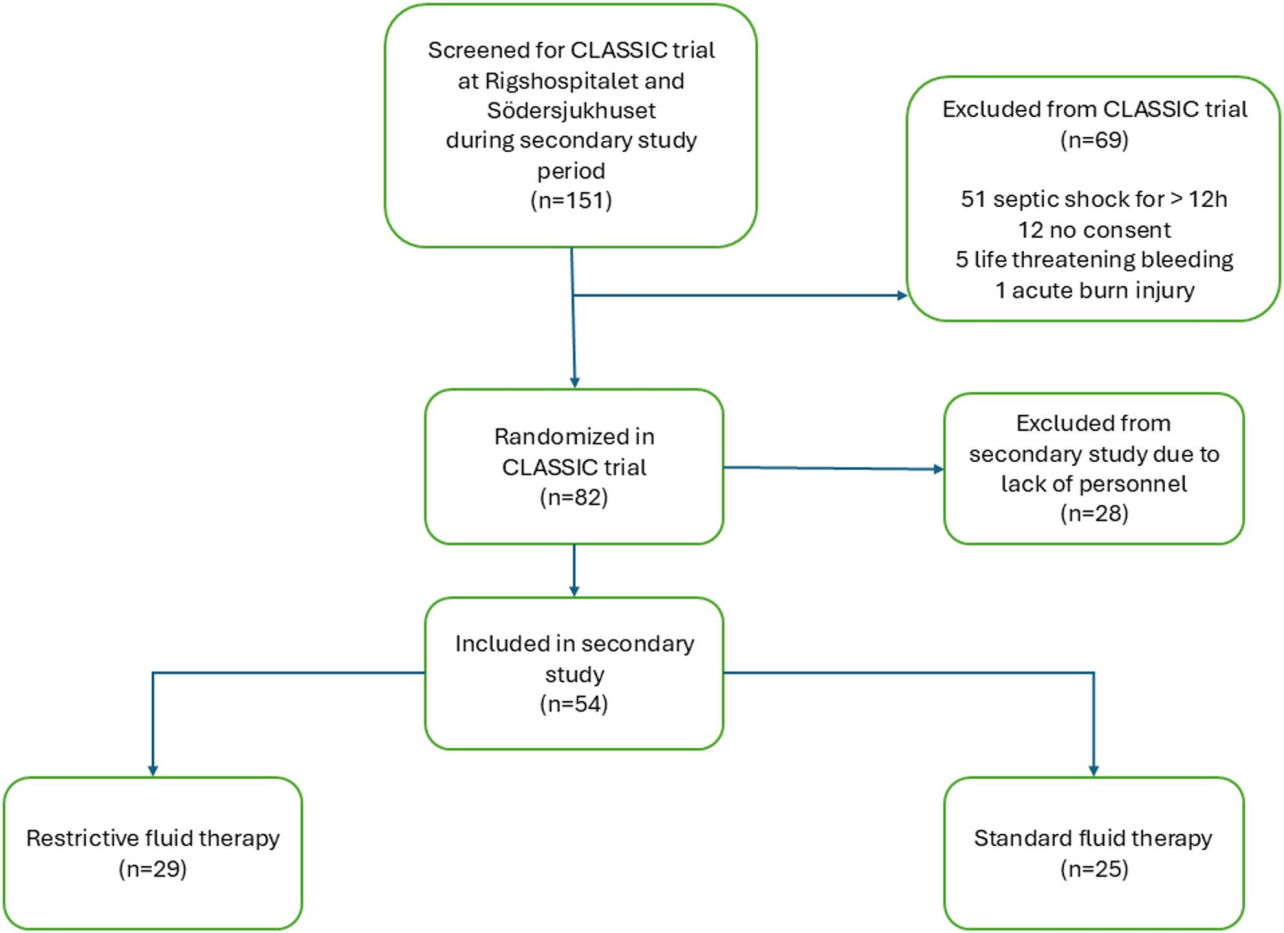
Screening, inclusion and randomisation. *Flow diagram showing screening*,* inclusion*,* exclusion and randomisation. This diagram is reproduced from a previously published CLASSIC sub-study* [25] *distributed under the Creative Commons Attribution (CC-BY) license*

Due to staffing constraints during the COVID-19 pandemic, patient recruitment for this sub-study progressed more slowly than anticipated. Therefore, the target sample size of 104 was not reached before the main CLASSIC trial was concluded. In total, 54 patients were enrolled: 14 at Södersjukhuset and 40 at Rigshospitalet (Fig. 1). Of these, 29 were assigned to the restrictive fluid group and 25 to the standard fluid group. Baseline characteristics, including age, sex and predicted 90-day mortality, were largely comparable between the groups (Table 1). However, GI-infection and use of respiratory support were more common in the restrictive fluid group, whereas preexisting ischemic heart disease or heart failure was more common in the standard fluid group (Table 1). The median interval from T0 to T1 was 16.8 h (IQR 10.8–21.0) in the restrictive group compared to 18.8 h (IQR 14.0–22.5) in the standard group. A total of 67 samples were missing across 43 patients, representing 31.0% of the total dataset. The distribution of missing samples was as follows; 18 at T0, 2 at T1, 13 at T2 and 34 at T3 (Table 1).

**Table 1:** Patient characteristics at baseline

Characteristics	Restrictive Fluid (*N* = 29)	Standard Fluid (*N* = 25)
Median age (IQR) — yr	72 (63.5–77.5)	72 (70.0–78.0)
Male sex — no. (%)	18 (62.1)	16 (64.0)
Coexisting condition — no. (%)		
Hematologic or metastatic cancer	9 (31.0)	6 (24.0)
Ischemic heart disease or heart failure	3 (10.3)	9 (36.0)
Chronic hypertension	13 (44.8)	12 (48.0)
Long-term dialysis †	1 (3.4)	3 (12.0)
Median time from ICU admission to randomisation (IQR) — hr	3.5 (1.6–12.3)	4.8 (1.1–13.4)
Median predicted 90-day mortality (IQR) — % ‡	23.0 (19.5–28.0)	24 (20.0–27.0)
Inclusion site — no. (%)		
Södersjukhuset IVA	6 (20.7)	8 (32.0)
Rigshospitalet	23 (79.3)	17 (68.0)
Source of ICU admission — no. (%)		
Emergency department or prehospital	9 (31.0)	5 (20.0)
Hospital ward	10 (34.5)	12 (48.0)
Operating or recovery room	9 (31.0)	7 (28.0)
Another ICU	1 (3.4)	1 (4.0)
Focus of infection — no. (%) §		
Gastrointestinal	10 (34.5)	5 (20.0)
Pulmonary	6 (20.7)	5 (20.0)
Urinary tract	4 (13.8)	8 (32.0)
Skin or soft tissue	5 (17.2)	3 (12.0)
Other	4 (13.8)	4 (16.0)
Body weight, blood values, and interventions		
Median body weight (IQR) — kg	79.0 (67.0–93.5)	78.0 (68.0–91.0)
Median highest plasma lactate (IQR) — mmol per liter ¶	3.5 (2.6–4.9)	3.9 (2.9–5.7)
Median highest dose of norepinephrine (IQR) — µg/kg/min ‖	0.28 (0.14–0.46)	0.25 (0.1–0.46)
Median volume of intravenous fluid 24 h before randomisation (IQR) — ml **	2811 (1486–4032)	2983 (2050–3988)
Use of systemic glucocorticoid — no. (%)	11 (37.9)	6 (24.0)
Median highest plasma creatinine (IQR) — mg/dl ††	147.0 (103.5–257.5)	154.0 (117.5–243.5)
Use of respiratory support — no. (%) ‡‡	13 (44.8)	8 (32.0)
Missing samples — no. (%)		
Any missing sample	25 (86.2)	18 (72.0)
Missing T0	14 (48.3)	4 (16.0)
Missing T1	2 (6.9)	0 (0)
Missing T2	5 (17.2)	8 (32.0)
Missing T3	20 (69.0)	14 (56.0)

* *ICU* Intensive care unit. *IQR* Interquartile range

† Long-term dialysis was defined as the use of hemodialysis (or hemofiltration) or peritoneal dialysis at least once a week before hospital admission

‡ The predicted 90-day mortality was calculated from the Simplified Mortality Score for the Intensive Care Unit

§ The listed location was the documented or suspected focus of infection at the time of randomisation

¶ Shown are the highest plasma lactate levels within the 3 h before randomisation

‖ The infusion rate of norepinephrine reflects the highest rate within the 3 h before randomisation

** Volumes of intravenous fluid within the 24 h before randomisation were defined as all crystalloid fluids, colloid fluids and blood products the patient had received within the 24 h before undergoing randomisation, independent of location (in-hospital or prehospital) and including intravenous fluids that contained medication or nutrition

†† Values reflect the highest plasma creatinine level within the 24 h before randomisation

‡‡ Respiratory support includes the continuous use of invasive or non-invasive mechanical ventilation or continuous positive airway pressure at baseline

There was no missing baseline data. This table is reproduced from a previously published CLASSIC sub-study [25], distributed under the Creative Commons Attribution (CC-BY) license

### Fluid characteristics and CLASSIC outcomes

Patients in the restrictive fluid group received less IV fluid (cumulative volumes of IV fluids administered in the ICU, excluding blood products, medication and nutrition) from randomisation through day 1, day 5 and ICU discharge/90 days (Table 2). The same trend, although more pronounced, was seen for cumulative fluid balance. Median total fluid volume given (IV fluids, blood products, nutrition, IV and oral medications, and oral fluid intake) was also lower in the restrictive group at day 1. However, the opposite was seen for total fluid volumes at 5 days and at ICU discharge/90 days, although the variability in these measurements was considerable (Table 2). In total, ≥ 1 IV fluid protocol violations were observed in 10 patients (34.4%) in the restrictive fluid group and 3 patients (12.0%) in the standard fluid group. The median ICU length of stay was 6 days (IQR 4–10) in the restrictive group compared to 4 (IQR 3–7) in the standard group. Ninety-day mortality was 48.3% in the restrictive fluid group versus 28.0% in the standard fluid group. A detailed summary of fluid types is presented in Supplementary Table 1.

**Table 2:** Cumulative fluid volumes and balances in millilitres

	Restrictive Fluid Group (*N* = 29)	Standard Fluid Group (*N* = 25)	Difference (Restrictive vs. Standard)	
Intravenous fluid volume†				
After 1 day‡				
Median (IQR)	320 (0–940)	926 (215–1887)	-606	
Mean	558	1,213	-655	
After 5 days				
Median (IQR)	1,420 (471–2,556)	2,379 (1,037–3,555)	-959	
Mean	2,163	2,941	-778	
At ICU discharge ‖				
Median (IQR)	1,795 (90–4377)	2,715 (1037–5600)	-920	
Mean	3,399	3,391	8	
Total fluid volume§				
After 1 day‡				
Median (IQR)	2,163 (834–3,382)	2,361 (1,514–3,907)	-198	
Mean	2,180	2,704	-524	
After 5 days				
Median (IQR)	10,601 (6,700–13,850)	8,066 (5,434–10,487)	2,535	
Mean	10,589	9,654	935	
At ICU discharge ‖	+			
Median (IQR)	13,644 (6,701–29,250)	8,066 (5,440–20,823)	5,578	
Mean	25,835	14,113	11,722	
Cumulative fluid balance¶				
After 1 day‡				
Median (IQR)	385 (41–1,145)	890 (483–2,364)	-505	
Mean	321	1,369	-1,048	
After 5 days				
Median (IQR)	646 (-1,204–2,679)	2,045 (1,209–4,763)	-1,399	
Mean	682	2,801	-2,119	
At ICU discharge ‖				
Median (IQR)	-215 (-1,502–3,246)	1,323 (385–4,763)	-1,538	
Mean	753	2,386	-1,633	

* *ICU* Intensive Care Unit. *IQR* Interquartile Range

† Cumulative volumes of intravenous fluids administered in the ICU (excluding blood products and intravenous fluids with medication and nutrition)

‡ From the time of randomisation to the next start of the 24-hour fluid chart used by the ICU

§ Amounts represent the volumes of total fluid intake, including intravenous fluids, blood products, nutrition, intravenous and oral medications, and oral fluid intake

¶ Amounts represent the total volume of fluid intake minus the total fluid output, including urinary output, fluid removed by renal replacement therapy, and other fluid output (e.g., bleeding, ascites, diarrhea, or drain losses)

‖ Up to 90 days

There was no missing fluid data. This table is reproduced from a previously published CLASSIC sub-study [25] distributed under the Creative Commons Attribution (CC-BY) license

### Outcome

**Fig. 2:**
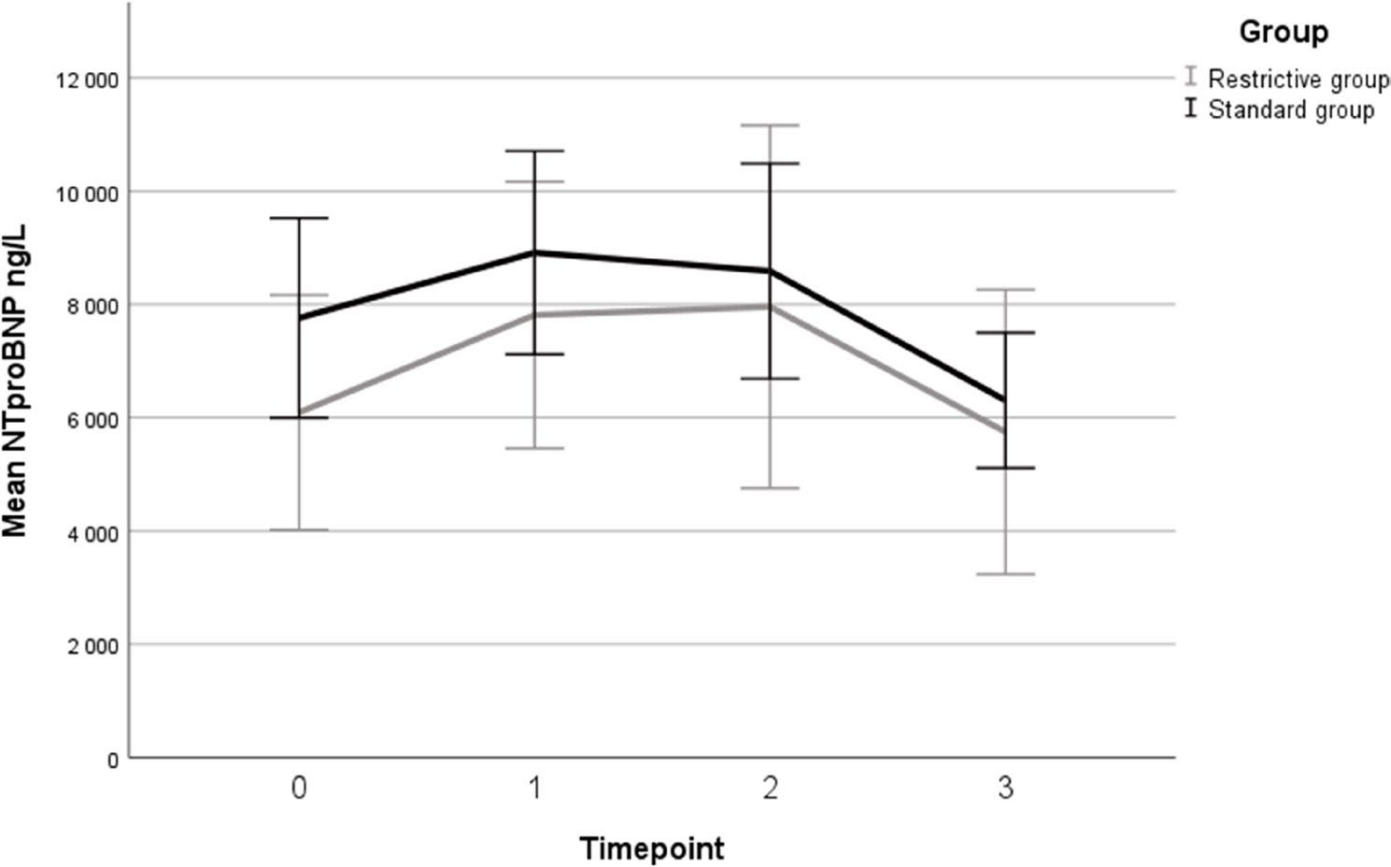
Plasma NTproBNP concentrations over time. *Graphical presentation of observed mean plasma NTproBNP concentrations across all timepoints. Number of cases at each timepoint: T0: 36*,* T1: 52*,* T2: 41*,* T3: 20. Error bars represent 95% confidence intervals*

**Fig. 3:**
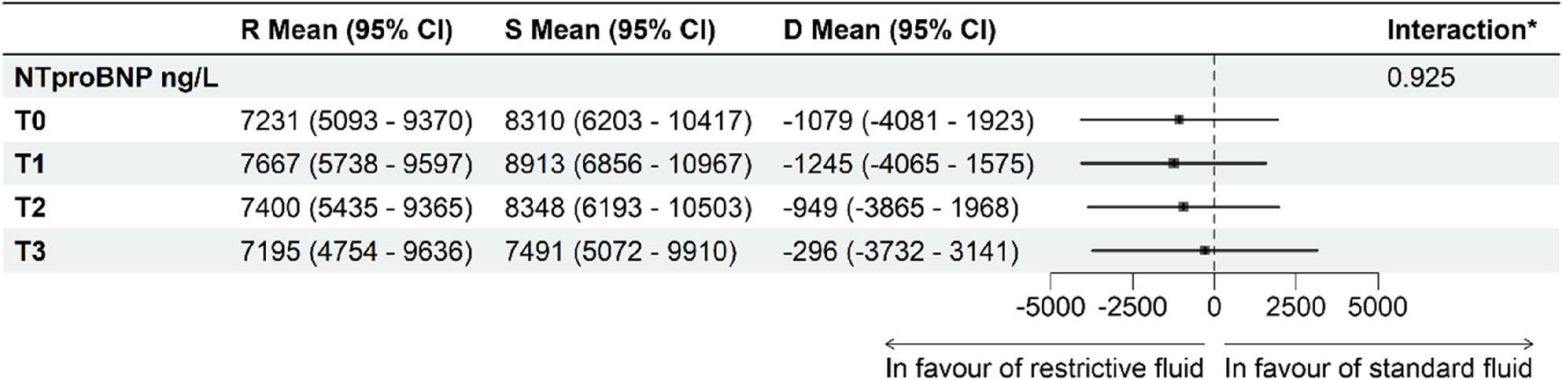
Between-group differences in NTproBNP over time. *Forest plot displaying differences in mean plasma levels of NTproBNP derived from mixed models analyses*, between the restrictive group and standard fluid group at each timepoint. R Mean: Mean plasma levels of NTproBNP in the restrictive fluid group. S Mean: Mean plasma levels of NTproBNP in the standard fluid group. D Mean: Differences in mean plasma levels of NTproBNP between the restrictive and standard fluid group. *P-value for interaction effect between group allocation and time across all timepoints

Mean levels of NTproBNP did not differ significantly between the standard fluid group compared to the restrictive fluid group at any timepoint (Figs. 2 and 3). At baseline (T0), mean NTproBNP was 7,231 ng/L (95% CI 5,093–9,370) in the restrictive group and 8,310 ng/L (95% CI 6,203–10,417) in the standard group, corresponding to a between-group difference of -1,079 ng/L (95% CI -4,081–1,923). Similar non-significant differences were observed at T1 (-1,245 ng/L (95% CI -4,065–1,575)), T2 (-949 ng/L, 95% CI -3,865–1,968) and T3 (-296 ng/L, 95% CI -3,732–3,141).

Likewise, no group-by-time interaction was observed (*p* = 0.925), indicating that temporal changes were similar in both groups (Fig. 3).

NTproBNP levels did not correlate significantly with cumulative fluid balance at either T1 (*r* = 0.07, *p* = 0.32) or T3 (*r* = − 0.03, *p* = 0.69), when analysed irrespective of treatment group (Supplementary Figs. 4 and 5).

## Discussion

### Main findings

In this sub-study of the CLASSIC trial, we found no significant differences in plasma NTproBNP levels between adult ICU patients with septic shock randomised to restrictive versus standard IV fluid therapy during ICU stay. However, the limited sample size and resulting likely low power of our study, precludes firm conclusions regarding causal relationships between fluid strategy, fluid balance and NTproBNP to be drawn from our data.

### Generalisability

Plasma levels of NTproBNP in our cohort were extremely elevated, consistent with previous studies on septic populations, with mean concentrations well above normal reference range (< 125 ng/L below age 75, < 450 ng/L above age 75) [13, 14]. Observational studies have described associations between greater positive net fluid balance and early rise in NTproBNP in critically ill patients [19].

Elevated NTproBNP in septic shock likely reflects a complex interplay between myocardial strain, systemic inflammation, catecholamine exposure, renal clearance and other confounding factors. It is possible that restrictive fluid therapy, while altering preload, exerts only a minor influence on overall NTproBNP concentrations in this setting. Indeed, NTproBNP levels are widely elevated in septic shock patients, with several studies showing inconsistent associations between cardiac function and NTproBNP in sepsis [15, 18, 26]. This suggests that inflammatory stimuli, rather than myocardial wall strain alone, may be the predominant driver of NTproBNP concentrations in this setting [27–29], possibly limiting its utility as a marker for cardiac load in septic shock. The absence of correlations between NT-proBNP and fluid balance in our cohort, irrespective of group allocation, further suggests that any relationship between fluid status and NT-proBNP may be weak.

In the present study, the two treatment groups were largely comparable regarding factors known to affect NTproBNP, including age, renal function and vasopressor exposure. However, some baseline imbalances were observed. Preexisting ischemic heart disease or chronic heart failure was more common in the standard fluid group, which could have increased susceptibility to NTproBNP release and may explain higher baseline NTproBNP levels in this group. This is consistent with studies linking pre-existing cardiac disease to elevated cardiac biomarkers in sepsis [30]. Conversely, GI-infection was more common in the restrictive fluid group; a condition typically associated with greater fluid losses and more severe septic shock. This may have influenced fluid administration unevenly and contributed to the higher use of respiratory support and 90-day mortality observed in the restrictive group. The greater overall disease burden in this group might also have contributed to increased NTproBNP concentrations. Worth noting is that in the CLASSIC trial no difference in 90-day mortality was seen between groups.

### Strengths and limitations

As a sub-study of a large international randomised trial, this study benefits from rigorous trial infrastructure. Despite a smaller than planned cohort, between-group differences in administered IV fluid volume, total fluid volume, and cumulative fluid balance were achieved at T1, indicating adherence to the protocol. Laboratory analyses were centralised to a single laboratory, and sampling followed a prespecified protocol, minimizing local variations.

There are several important limitations to our study. Group allocation was not blinded to staff or patients. A lack of research personnel due to the coinciding COVID-19 pandemic resulted in a final sample size of 54 patients rather than the preplanned 104, with relatively large amounts of missing data recorded. Both groups had received considerable volumes of intravenous fluids prior to randomisation (median > 2800 ml), which may have contributed to elevated baseline NT-proBNP levels and reduced sensitivity to detect effects of the assigned fluid strategy. Although the restrictive group received less IV fluid and displayed a more negative total fluid balance compared with the standard group throughout the study period, the magnitude of these differences (-606 mL – -1538 mL) may have been too small to meaningfully affect cardiac load. It is also worth noting that the volume of study-assigned fluid administered constituted a relatively small proportion of total fluid volume received, a common occurrence in the ICU setting potentially due to “fluid creep”, which may have diluted any potential effect of the restrictive fluid strategy [31]. The larger median volume of total fluids observed in the restrictive group compared to the standard group at 5 days and ICU discharge/90 days may reflect the above-mentioned higher incidence of GI infection in the restrictive fluid group, with subsequent larger fluid requirements and longer ICU stay. Finally, no objective measurements of cardiac output were collected, limiting our ability to directly assess cardiac function and interpret the mechanisms behind the observed NTproBNP patterns.

## Conclusions

In this exploratory sub-study of the CLASSIC trial, we found no statistically significant difference in plasma NTproBNP between adult ICU patients with septic shock randomised to restrictive versus standard IV fluid therapy. The absence of group differences, together with uniformly elevated NTproBNP levels across treatment groups, could suggest that NTproBNP elevation in septic shock primarily reflects an inflammatory state rather than fluid induced cardiac loading. Given the small sample size of this study, these results should be interpreted with caution.

## Supplementary Information

Supplementary Material 1.

Supplementary Material 2.

Supplementary Material 3.

## Data Availability

The datasets used and/or analysed during the current study are available from the corresponding author on reasonable request.

## Abbreviations

AR(1): First-order autoregressive covariance structure
BMI: Body mass index
CC BY: Creative Commons Attribution
CI: Confidence interval
CLASSIC: Conservative versus Liberal Approach to Fluid Therapy of Septic Shock in Intensive Care
COVID-19: Coronavirus disease 2019
EDTA: Ethylenediaminetetraacetic acid
ELISA: Enzyme-linked immunosorbent assay
GI: Gastrointestinal
hsTnT: High-sensitivity troponin T
ICU: Intensive care unit
IQR: Interquartile range
IV: Intravenous
NTproBNP: N-terminal pro-B-type natriuretic peptide
SCM: Septic cardiomyopathy
