# Supplementary material for: Restrictive versus standard intravenous fluid therapy and NTproBNP in ICU patients with septic shock – a sub-study of the randomised CLASSIC trial
Source: BMC Anesthesiol. 2026 Apr 15;26:241. doi: 10.1186/s12871-026-03836-6 (PMC13085469; doi:10.1186/s12871-026-03836-6)
Supplement: Supplementary file 1 — Supplementary Material 1. [file 12871_2026_3836_MOESM1_ESM.docx]

**Figure 4**. Correlation between NTproBNP and fluid balance at T1.
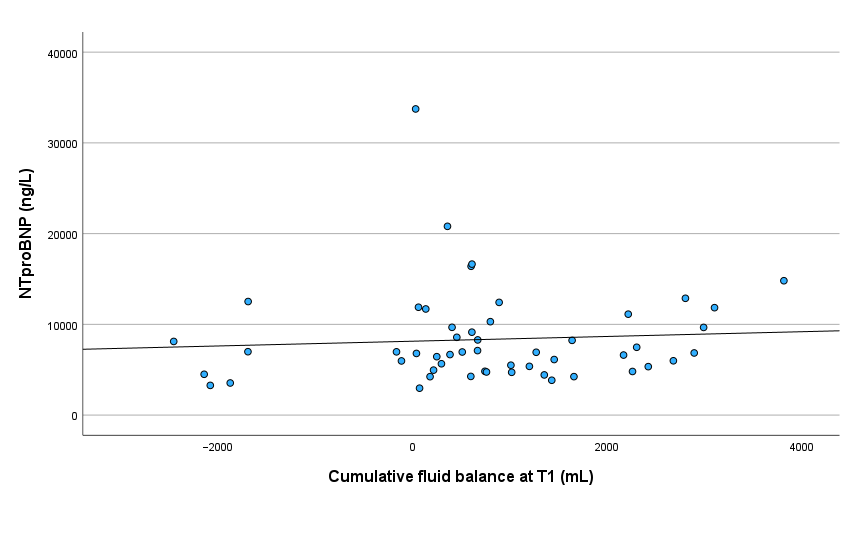


*Scatter plot illustrating the relationship between cumulative fluid balance at T1 and NTproBNP concentrations. Each point represents an individual observed patient measurement. The solid line represents a fitted linear regression line based on ordinary least squares.*

**Figure 5**. Correlation between NTproBNP and fluid balance at T3.


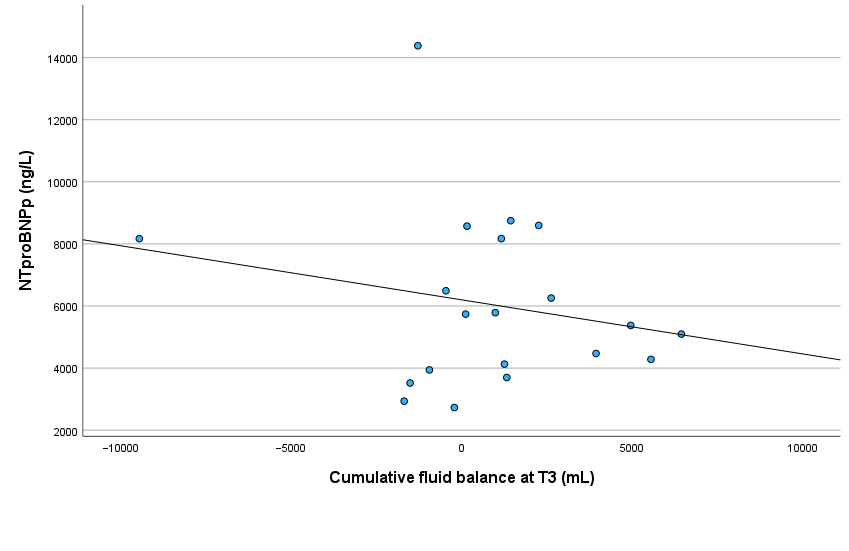


*Scatter plot illustrating the relationship between cumulative fluid balance at T3 and NTproBNP concentrations. Each point represents an individual observed patient measurement. The solid line represents a fitted linear regression line based on ordinary least squares.*
