# Supplementary material for: Restrictive versus standard intravenous fluid therapy and NTproBNP in ICU patients with septic shock – a sub-study of the randomised CLASSIC trial
Source: BMC Anesthesiol. 2026 Apr 15;26:241. doi: 10.1186/s12871-026-03836-6 (PMC13085469; doi:10.1186/s12871-026-03836-6)
Supplement: Supplementary file 2 — Supplementary Material 2. [file 12871_2026_3836_MOESM2_ESM.docx]

**Supplementary table** Fluid characteristics

|  | **Restrictive Fluid Group (N=29)** | **Standard Fluid Group (N=25)** | **Difference (Restrictive vs. Standard)** |
| --- | --- | --- | --- |
| **Type of fluid** |  |  |  |
| Ringer’s acetate |  |  |  |
| Median (IQR) | 0 (0 – 200) | 0 (0 – 200) | 0 |
| Mean | 808 | 438 | 370 |
| Ringer’s lactate |  |  |  |
| Median (IQR) | 0 (0 – 1000) | 0 (0 – 911) | 0 |
| Mean | 673 | 358 | 315 |
| Isotonic saline |  |  |  |
| Median (IQR) | 50 (0 – 645) | 2 (0 – 500) | 48 |
| Mean | 557 | 878 | -321 |
| Albumin |  |  |  |
| Median (IQR) | 200 (0 – 700) | 100 (0 – 728) | 100 |
| Mean | 572 | 439 | 133 |
| Blood products* |  |  |  |
| Median (IQR) | 360 (0 – 1815) | 0 (0 – 485) | 360 |
| Mean | 1111 | 272 | 839 |
| Other fluids | + |  |  |
| Median (IQR) | 70 (0 – 900) | 1110 (0 – 1942) | -1040 |
| Mean | 787 | 1278 | -491 |

| *IQR = interquartile range.*  ** Total transfused volume of blood components, defined as red blood cells, plasma, and platelet concentrates.* |
| --- |
| *† Total volume of other IV fluids: e.g. <10% glucose, glucose-potassium, sodium-potassium-chloride, half-saline.*  *This table is reproduced from a previously published CLASSIC sub-study (23)* *distributed under the Creative Commons Attribution (CC-BY) license.* |
